# A classification system for describing N‐fertilizer performance in dryland wheat crops of the inland Pacific Northwest

**DOI:** 10.1002/jeq2.70017

**Published:** 2025-03-25

**Authors:** Joaquin J. Casanova, David R. Huggins, Claire L. Phillips

**Affiliations:** ^1^ USDA ARS NSAR Pullman Washington USA

## Abstract

Wheat (*Triticum aestivum* L.) crops in the inland Pacific Northwest demand nitrogen (N) fertilizers at high levels to achieve yield and grain protein objectives. Inefficiencies in N use can accelerate soil acidification, contribute to N_2_O emissions, and result in unnecessary input costs. Reducing N losses is a complicated problem, as producers have to consider grain protein and yield targets, co‐limitations of water and other nutrients, longer term soil health goals, and variability in crop performance across fields. However, past work in the region has established that there are at least four prevalent N performance syndromes, each of which have different environmental effects and lend themselves to different actions for adapting N management. In this paper, we build on this work to develop a discrete six‐class evaluation system that simplifies assessment of wheat N performance. We use over 20 years of harvest data from the Cook Agronomy Farm Long‐Term Agroecosystem Research site to assess spatial and temporal patterns in wheat N performance. While some areas had durable high or low nitrogen performance, there was year‐to‐year variation due to weather, management, and cultivar‐specific factors. For wheat management decisions, the categorical system narrows the range of possible problems, potential environmental effects, and solutions to poor wheat performance.

## Core Ideas

- There is a need to better quantify and explain reasons for nitrogen efficiency in wheat.
- A six‐class performance ranking system considers cultivar class, protein concentration, grain yield, and nitrogen use.
- Wheat at a long‐term agricultural research farm, over 20 years, are examined using the performance classes to highlight areas with nitrogen losses.

**Abbreviations**
CAF: Cook Agronomy Farm
GPC: grain protein concentration
HRSW: hard red spring wheat
HRWW: hard red winter wheat
iPNW: inland Pacific Northwest
NBI: nitrogen balance index
SWSW: soft white spring wheat
SWWW: soft white winter wheat

## 1 INTRODUCTION

Nitrogen (N) fertilizer poses a tremendous challenge for modern agriculture, as it is essential to support crop yields but has major environmental and human health costs (Sobota et al., 2015). As N fertilizer prices rise globally (McConnell et al., 2022; Nti, 2022), economic pressures add to the imperative to use N effectively and efficiently. The inland Pacific Northwest (iPNW) dryland cropping system region is one of the United States' largest producers of wheat (*Triticum aestivum* L.). Effective N management is particularly challenging in this region because the Mediterranean climate provides little in‐season precipitation, and in‐crop N applications are therefore not transported effectively into the profile. Growers rely heavily on past performance to make N management decisions, as well as assimilating diverse information about costs, weather, market demands on cultivar selection, and weighing long‐term soil health goals (Pan et al., 2017). Given the complexities they face, growers report being interested in ways of evaluating the performance of their management decisions.

Wheat producers in the iPNW can judge their management decisions by several factors, including grain yield, input use efficiency, and grain protein concentration (GPC), which effects grain value. Soft white wheat (both spring and winter; referred to here as soft white spring wheat and soft white winter wheat [SWWW], respectively) are low protein wheats used for cookies, crackers, cakes, and flatbreads (US Wheat Associates, 2023). Hard red wheat (both spring and winter; referred to here as hard red spring wheat [HRSW] and hard red winter wheat [HRWW], respectively) require higher protein levels for bread baking (US Wheat Associates, 2023). Buyers apply discounts to both wheat classes if optimal protein concentrations are not met. Along with choice of cultivar and crop rotation, farmers' primary tools for manipulating wheat yield and protein concentration performance are N application rate, timing, placement, and chemical form. Because protein is approximately 16% N by mass, N fertilizer directly influences GPC (Barneix, 2007).

Current N fertilizer recommendations focus only on reaching yield and protein goals, without considering the effects of N uptake efficiency (NUpE; aboveground N divided by N supply) and N utilization efficiency (NUtE; grain yield divided by aboveground N), which may vary spatially and temporally (D. R. Huggins & Pan, 1993, 2003). Field variations in these efficiency factors result in different yield and protein outcomes of current N recommendations. Growers in the iPNW are increasingly interested in evaluating the efficiency of their N use, due to high input costs and concerns about soil health, particularly long‐term soil acidification (Mahler et al., 1985; McFarland et al., 2020). The use of nitrogen balance index (NBI; defined here as grain N/fertilizer N; D. Huggins et al., 2010) is a readily measured metric of N‐use efficiency that is increasingly recommended for tracking agricultural N pollution (Eagle et al., 2020; McLellan et al., 2018). NBI is useful as an indicator for several reasons: it is easy to calculate from fertilizer rates, yield, and protein data; can be calculated and compared across diverse production systems; and can be related to N losses via N_2_O emissions and NO_3_^−^ leaching across broad geographic regions (Brown, 2015; Glover, 2018; McLellan et al., 2018; Taylor, 2016). Other methods based on the idea of a “safe operating space” (EU Nitrogen Expert Panel, 2015) use N input and N output for evaluating environmental effects but neglect cultivar or even crop type differences in setting boundaries. The use of a ratio as a metric allows better comparison, as the N required for hard red wheat is very different than that of soft wheat, for example.

However, NBI alone does not provide sufficient information to allow a producer to evaluate the effectiveness of their own N‐management decisions. Here, we describe an approach to evaluate N‐performance for dryland wheat production in the iPNW that incorporates not only whether a grower's production goals were met for yield and GPC, but also N efficiency. Using NBI, grain protein, and yield in combination gives a picture of how efficiently the crop was grown, its environmental effects, and how much viable product can be produced that meets market demands. This approach allows identification of areas of a field where N management could be adjusted to improve economic and environmental impacts. This system is based on a dichotomous key approach presented in D. Huggins et al. (2010) with several improvements. The previous classification system did not incorporate soft wheat varieties. Further, it is more accessible to growers as it only requires GPC, yield and applied N to assess performance, without needing crop residue or soil nitrogen. Tamagno et al. (2024) criticized ignoring soil N in assessing performance, a valid concern at a regional level. As a field‐scale tool for understanding management actions, the categorical system presented here helps focus on specific causes of poor nitrogen performance for individual management decisions.

**Core Ideas**

- There is a need to better quantify and explain reasons for nitrogen efficiency in wheat.
- A six‐class performance ranking system considers cultivar class, protein concentration, grain yield, and nitrogen use.
- Wheat at a long‐term agricultural research farm, over 20 years, are examined using the performance classes to highlight areas with nitrogen losses.

The objective of this work is to examine a categorical system for grading wheat performance. We use this system to look at historical performance at a trial at the R.J. Cook Agronomy Farm Long‐Term Agroecosystem Research (CAF‐LTAR) site, described in Section 2. We examine performance generally and describe the performance classes in relation to GPC, NBI, and yield. Then, we look at CAF‐LTAR spatially over time to demonstrate how to apply the method to understand N performance and the environmental impacts. Finally, in Section 3, we discuss utility of the classification method to growers, summarize how to interpret different classes, and detail future directions for developing a decision‐support tool.

## 2 MATERIALS AND METHODS

### 2.1 Cook Agronomy Farm

#### 2.1.1 Site description

**FIGURE 1:**
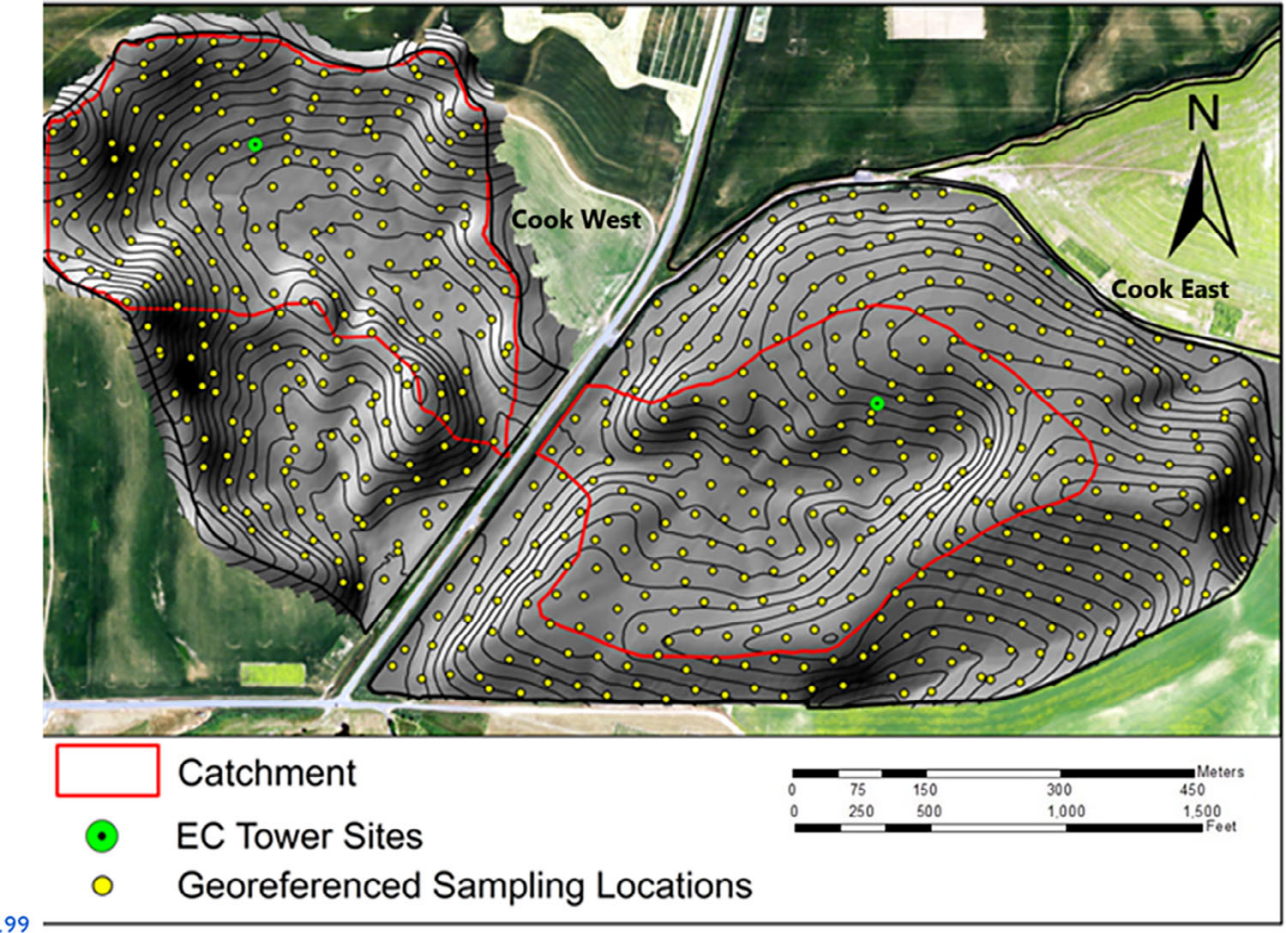
R.J. Cook Agronomy Farm (46°47′ N, 117°5′ W, 770 m above mean sea level) northeast of Pullman, WA. Yellow points show annually hand‐harvested locations. EC, eddy covariance tower site.

The R. J. CAF (Figure 1), north of Pullman, WA, is a 60‐ha site operated as part of the USDA LTAR network (Kleinman et al., 2018), with a comprehensive meteorological, harvest, and soil dataset (D. R. Huggins et al., 2024). Cook East, established in 1998, is a 37‐ha field that has been managed with no‐till practice (referred to as ALT or Alternative). Cook West, which was added in 2017, is a 23‐ha field that has been managed with conservational tillage (referred to as PRV or Prevailing).

**FIGURE 2:**
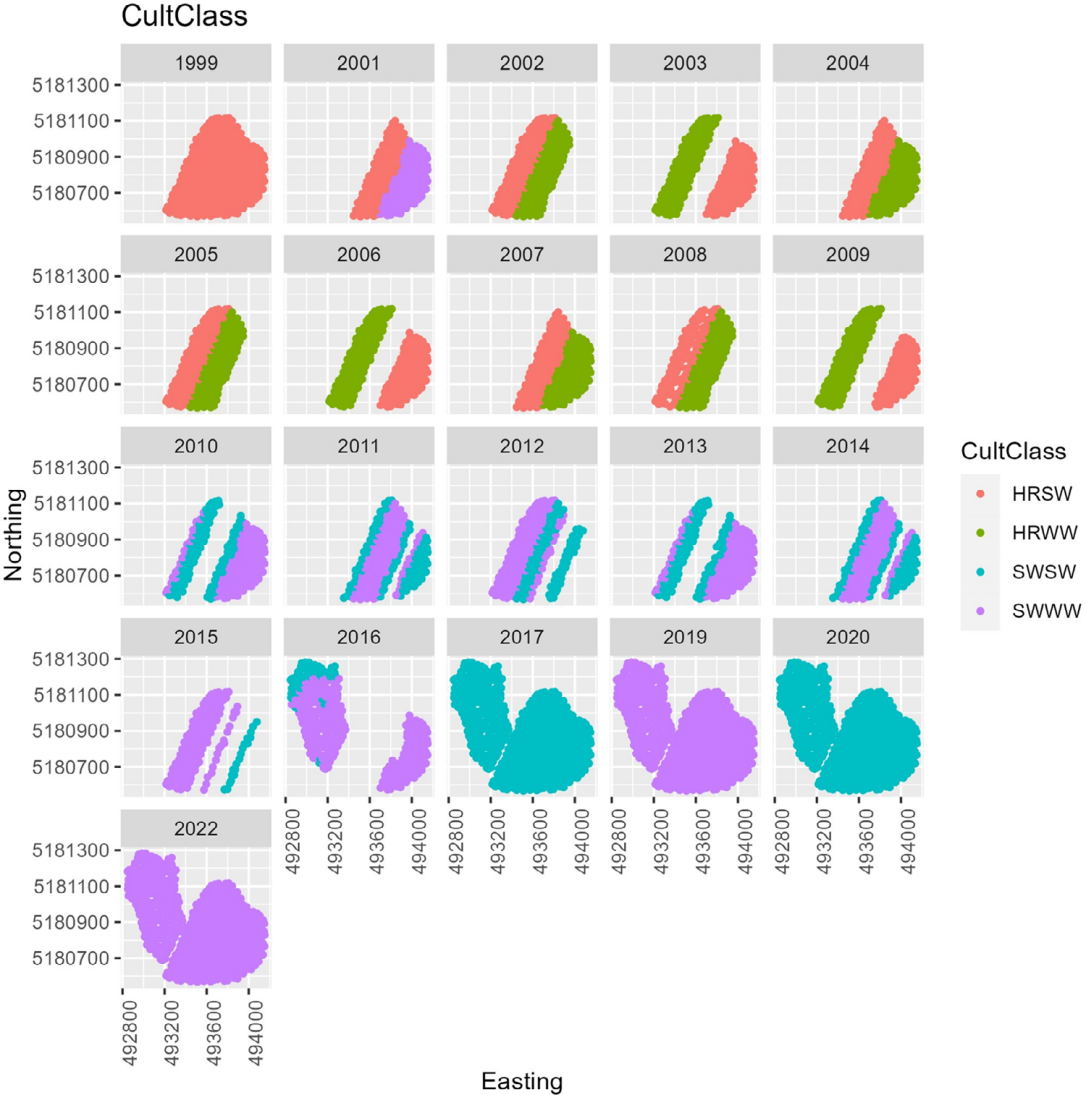
Cultivar class (hard red spring wheat [HRSW] vs. hard red winter wheat [HRWW] vs. soft white spring wheat [SWSW] vs. soft white winter wheat [SWWW]) planted each year and in what location over Cook Agronomy Farm (CAF). Axes are northing (*y*) and easting (*x*) in UTM (Universal Transverse Mercator) zone 11N. Years from 1999 to 2009 included primarily hard red varieties of wheat, with greater N demands. Years from 2010 to 2022 included entirely soft white wheat, with lower protein and lower N requirements. Crop on Cook West, 2016, was derived from the Cropland Data Layer.

Prior to 2017, CAF East was divided into sections with different rotations and fertilizer rates. Since 2017, both fields have been planted to a single crop every year, with CAF East split into high fertilizer and low fertilizer zones, whereas CAF West received one uniform rate. Crop sequences have varied over the site's history, and research has focused on evaluating potential crops to grow in rotation with wheat. Crop sequences included winter wheat grown in rotation with spring wheat, spring barley (*Hordeum vulgare*), canola (*Brassica napus annua* Koch), garbanzos (*Cicer arietinum*), lentils (*Lens culinaris*), and field peas (*Pisum arvense* L.). However, in this paper, we only examine portions of the field and years in which wheat was grown. Figure 2 shows the wheat years and the cultivar classes in each year.

The soil types are mostly Thatuna (a fine–silty, mixed, superactive, mesic Oxyaquic Argixeroll), Palouse (a fine–silty, mixed, superactive, mesic Pachic Ultic Haploxeroll), and Naff (a fine–silty, mixed, superactive, mesic Typic Argixeroll) (NRCS, 2023). The region has a Mediterranean climate with mean annual temperature of 9°C, rainfall of 518 mm, and snow of 831 mm (NOAA, 2023).

#### 2.1.2 Harvest and management data

A systematic grid of 369 georeferenced locations in CAF East and 250 locations in CAF West was established for annual sampling. At each location, crops were hand‐harvested from a 2 m × 1 m area. Plants were air dried and threshed to get total dry biomass and grain yield per area (G_y_). GPC and grain N (N_g_) were also measured with a Costech ECS4010 Elemental Analyzer and/or FOSS Infratec 1241 Grain Analyzer after finely milling the samples. Management data also included applied fall N (N_f,fall_) and spring N (N_f,spring_), planting and harvest dates, and cultivar class for each crop year.

#### 2.1.3 Topography and weather data

A Trimble RTK 4400 was used to collect high‐accuracy elevation data. Topographical variables were calculated from the DEM using R (R Core Team, 2016) with the package RSAGA (Brenning, 2008) at four scales (10, 30, 60, and 120). From this, other topographic variables were derived. Four features (potential incoming solar radiation, topographic wetness index, slope, and profile curvature) at the four scales were scaled for a total of 16.

Daily weather data were compiled from a nearby weather station (PULLMAN 2 NW). Variables included daily minimum and maximum temperatures, total precipitation, and snowfall, which was converted into precipitation (NOAA Severe Storms Laboratory, 2023). Maximum and minimum temperatures were averaged to estimate daily average. These daily values were aggregated into maximum, minimum, and average temperature (*T*_max_, *T*_min_, and *T*_ave_) over the growing season. Precipitation was aggregated into three even periods of 4 months: total precipitation 8–12 months before harvest (Precip_1_), 4–8 months before harvest (Precip_2_), and 4 months until harvest (Precip_3_).

### 2.2 Nitrogen performance classification

**FIGURE 3:**
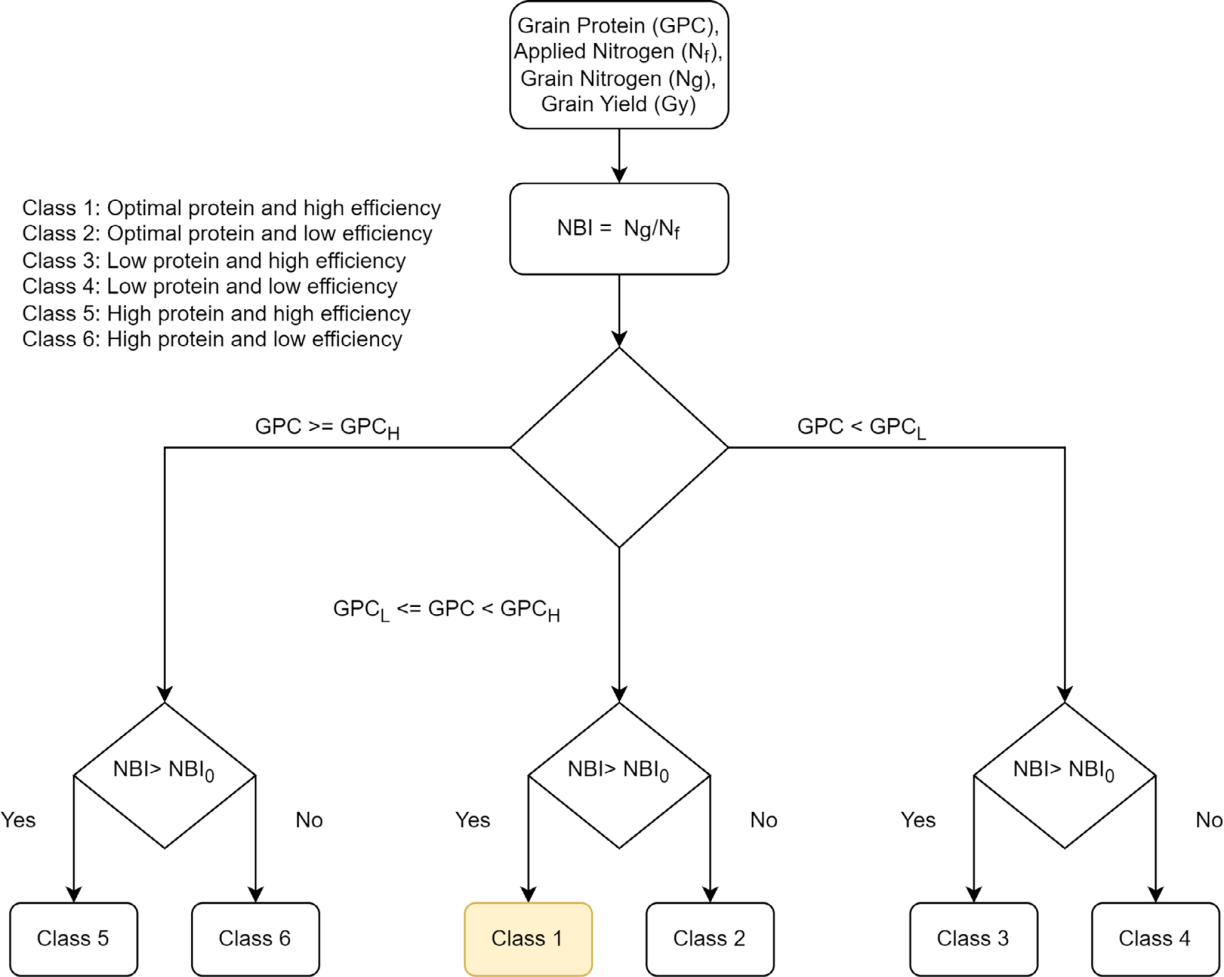
Decision tree for categorizing points into performance classes. GPC_H_ and GPC_L_ are high and low thresholds for GPC (where GPC is grain protein concentration). NBI_0_ is a threshold above which N balance index is considered efficient.

The CAF harvest dataset was used to classify the performance of each georeferenced point in each wheat growing season. Figure 3 shows the decision tree used to categorize points. For a given point, the protein level is classified based on cultivar class. If it is above an upper threshold, GPC_H_, or below a lower threshold, GPC_L_, the grain is discounted. Thresholds for GPC are based on market grades for cultivar classes (US Wheat Associates, 2023; USDA FSA, 2020), as shown in Table 1. For soft white wheat, GPC is ideally between the high and low thresholds to get the best price. Additionally, it is assumed that yield goals are met at the lower GPC threshold. For hard red wheat, there is effectively no GPC_H_ because higher protein concentration is desirable for bread making, so GPC_H_ is set arbitrarily high. After classifying by GPC, each point is classified by NBI. Above a cultivar‐dependent threshold (NBI_0_), fertilizer‐N use is considered efficient, and below a cultivar‐dependent threshold, fertilizer‐N use is considered inefficient. NBI_0_ was established based on examination of historical CAF data and a review of the literature (D. R. Huggins & Pan, 1993; Makowski et al., 1999), which suggest using an NUpE of 0.5 as a goal, which is built into fertilizer recommendations (Koenig, 2013). For the CAF dataset, we specifically used the median values of NBI over 1999–2023 as NBI_0_. When applying this classification approach to other locations, NBI_0_ can be considered a user‐defined parameter based on preference or the site‐specific historical performance. Other sources consider different thresholds (Brown, 2015; Glover, 2018; Taylor, 2016). The advantage of using the median on a long‐term dataset such as this is that it better highlights regions in the field with different performance, whereas an overly optimistic threshold places the bulk of the field into poorly performing classes, hiding spatial patterns. This NBI threshold will be highly dependent on the field site and management history.

**TABLE 1:** Thresholds used in wheat performance classification.

Cultivar class	NBI_0_ (‐)	GPC_L_ (‐)	GPC_H_ (‐)
HRWW	0.48	0.115	0.250^a^
HRSW	0.41	0.140	0.250^a^
SWWW	0.55	0.095	0.105
SWSW	0.74	0.095	0.105

*Note*: GPC_H_ and GPC_L_ are high and low thresholds, respectively, for GPC (where GPC is grain protein concentration). NBI_0_ is a threshold above which N balance index is considered efficient.

Abbreviations: HRSW, hard red spring wheat; HRWW, hard red winter wheat; SWSW, soft white spring wheat; SWWW, soft white winter wheat.

^a^ Hard red wheat is a cultivar class used for bread, with no upper threshold for protein concentration. GPC_H_ was assigned an arbitrary high value.

The final N performance classes can be described as follows:

- Class 1: Optimal protein and efficient N use (the ideal).
- Class 2: Optimal protein and inefficient N use.
- Class 3: Low protein and efficient N use.
- Class 4: Low protein and inefficient N use.
- Class 5: Excessive protein and efficient N use.
- Class 6: Excessive protein and inefficient N use.

Classes 5 and 6, in which grain protein levels are too high, effectively do not occur in hard varieties of wheat. Lower protein typically also means that yield targets were not met.

While Class 1 is clearly the ideal class, the hierarchy of remaining classes depend on priorities. Prioritizing protein goals over fertilizer efficiency would mean the class order 1–6 is the same as the order of preference. Alternately, prioritizing fertilizer efficiency, and thus environmental impacts, over protein and yield goals would imply that the next best class is 3. A full description of potential causes, effects and remedies for each class is given in Section 3.

## 3 RESULTS AND DISCUSSION

### 3.1 General relationships between classes and N‐performance

**FIGURE 4:**
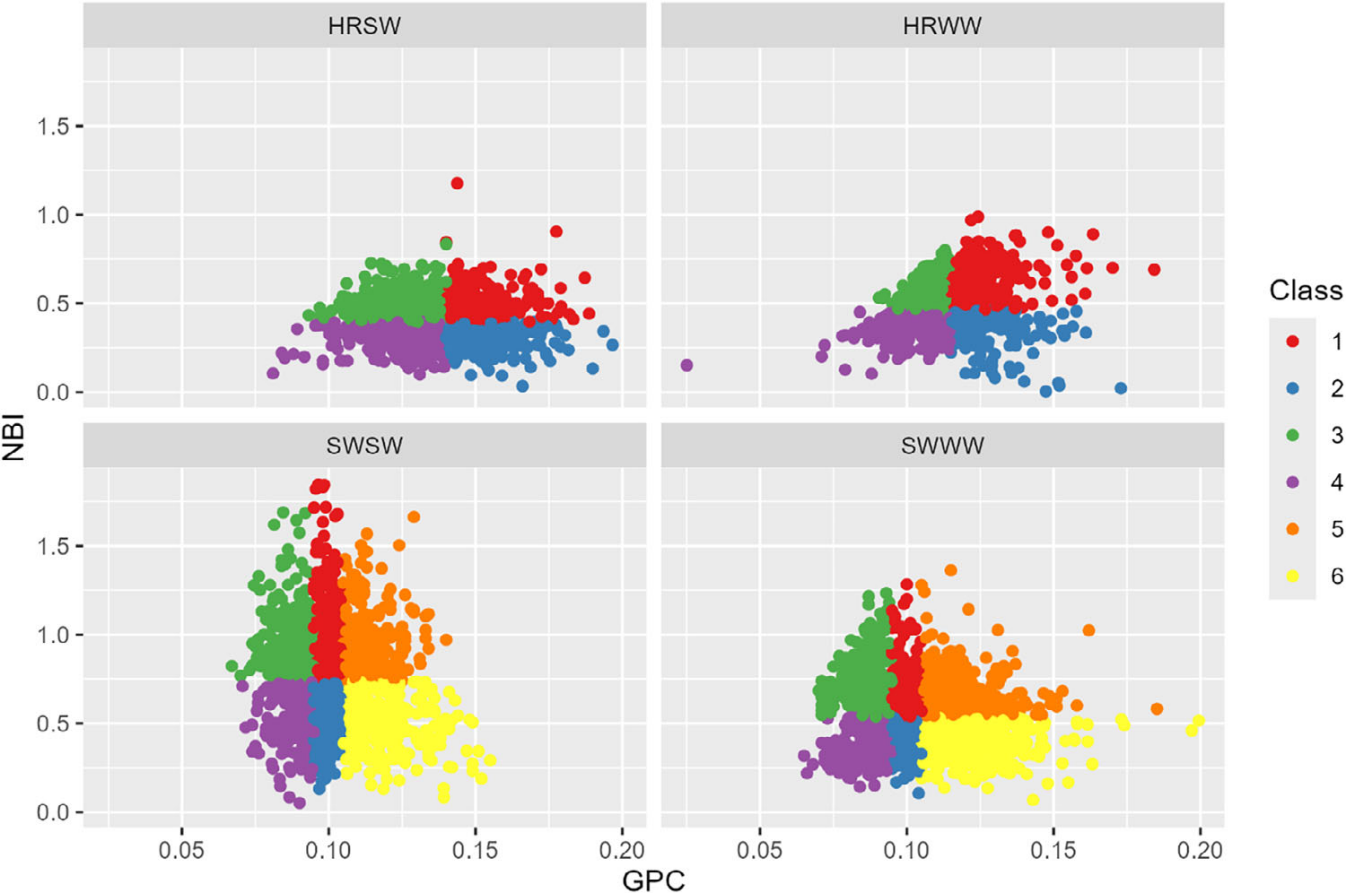
N balance index (NBI) versus grain protein concentration (GPC), demonstrating the classification system. HRSW, hard red spring wheat; HRWW, hard red winter wheat; SWSW, soft white spring wheat; SWWW, soft white winter wheat.

**FIGURE 5:**
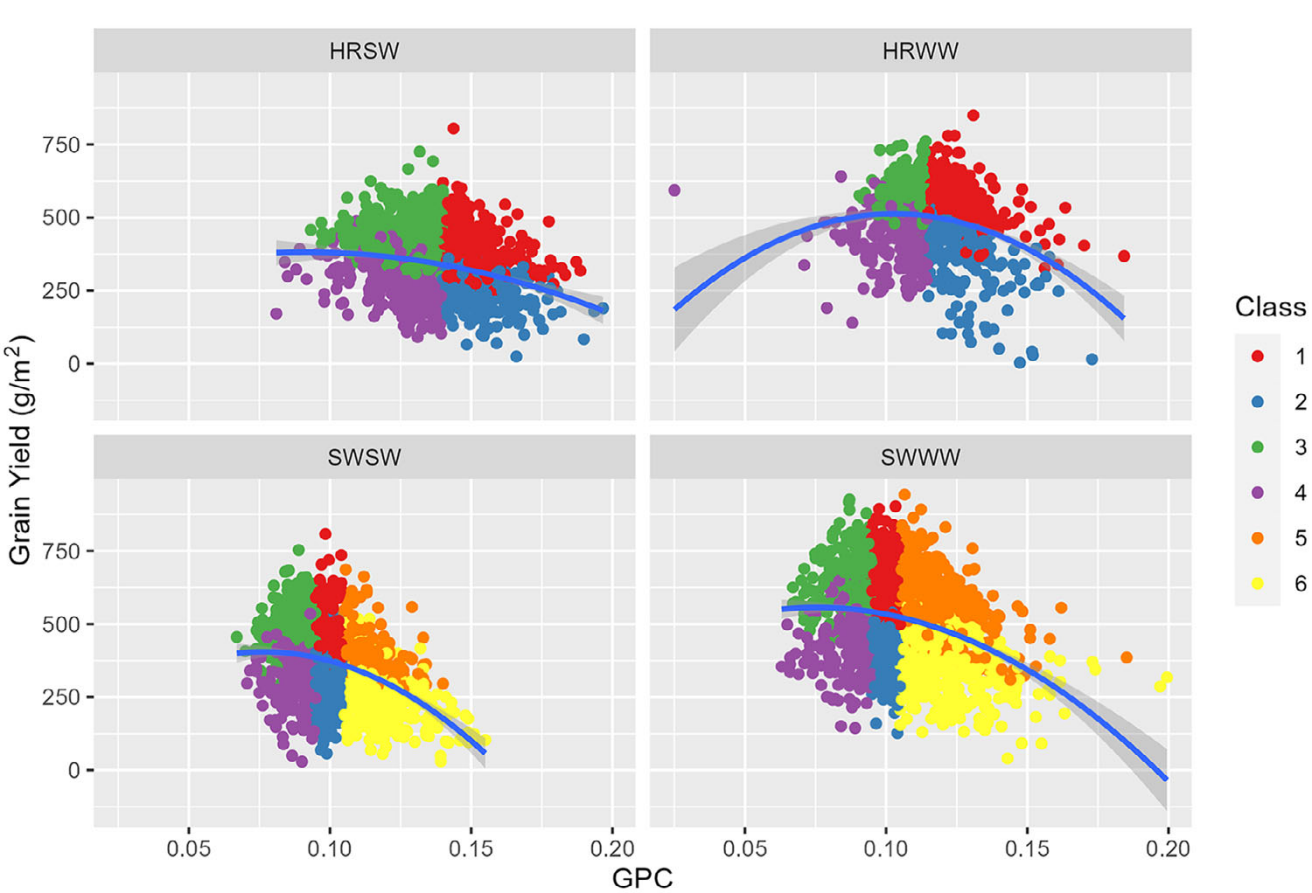
Grain yield versus grain protein concentration (GPC), demonstrating the classification system and quadratic response. HRSW, hard red spring wheat; HRWW, hard red winter wheat; SWSW, soft white spring wheat; SWWW, soft white winter wheat.

**FIGURE 6:**
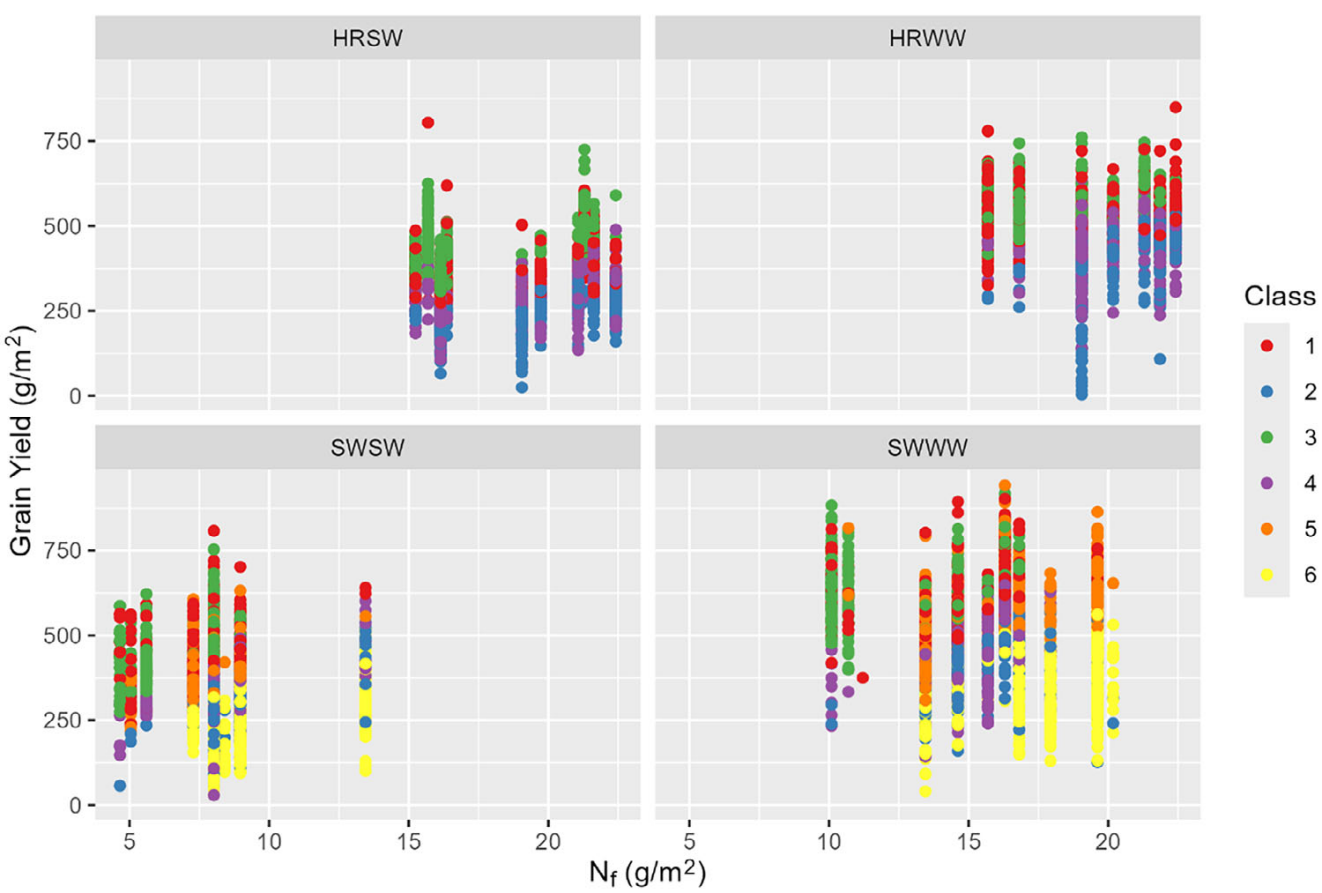
Grain yield versus applied N (N_f_). HRSW, hard red spring wheat; HRWW, hard red winter wheat; SWSW, soft white spring wheat; SWWW, soft white winter wheat.

Examining the trends in GPC, NBI, grain yield, and applied N simultaneously (Figures 4, 5, 6), we see the different cultivar classes form regions in the NBI versus GPC plane. NBI had a greater spread in values for soft wheat varieties, reflective of the greater range of N_f_ values and a greater spread of protein values. For higher NBI, we observe slightly lower GPC in soft varieties. In Figure 5, we observe a quadratic response, demonstrating peak yield is achieved at a particular protein level. Growers reducing N_f_ may not achieve potential yield, but peak yield generally falls within the protein concentration range favored by the market, though for soft wheat, yield may be higher at higher protein. With regard to applied N, the grain yield (Figure 6) tended to increase slightly with increasing N_f_, with a large amount of variability within each class. This indicates the different N responses in the field over space and time, seen also in Glover (2018). Grain yield response shows distinct clusters of the classes, which tended to follow a trend similar to those in T. Maaz and Pan (2017), who studied Louise, a SWWW cultivar. Classes 1, 3, and 5 have higher yield, and Classes 2, 4 and 6 have lower efficiency at the same GPC. However, there do exist some situations in which the more efficient class has lower yield. For example, low yielding Class 5 could occur if temperature, moisture, pests, or other nutrients limited grain carbohydrate accumulation but there was sufficient N for grain protein, leading to higher GPC. It also indicates that if yield was not limited by those other factors, that gains in N efficiency could be realized by reducing applied N.

The scatter of yield versus GPC is due to the spatial variability of potential yield. This variation in response could be due to topography, cropping history, and residual fertilizer (Glover, 2018; T. Maaz & Pan, 2017), and this spatial variability in potential yield must be considered when applying N and assessing other crop stress. Examining the trends in Plots 8–10 together, higher N_f_ rates led to excessive protein and lower yields in Class 6, whereas Class 4 experienced N_f_ losses or had other limiting factors, leading to low protein and low yield. In these two cases, there is a clear corrective measure to achieve better performance. Class 6 could reduce N_f_, thereby reducing protein to move into a more preferable class with higher NBI and lower GPC. Class 6 may also be symptomatic of other stresses, with high protein and low yield, it could indicate that yield was limited by other factors. Applying more N to Class 4 could lead to greater amounts of lost N to leaching or emissions if it occurs in a portion of the farm which is historically inefficient, and further description of management on environmental impacts by class is provided in Section 3.3.

### 3.2 Spatiotemporal trends in NBI and GPC, and N‐performance class at CAF

**FIGURE 7:**
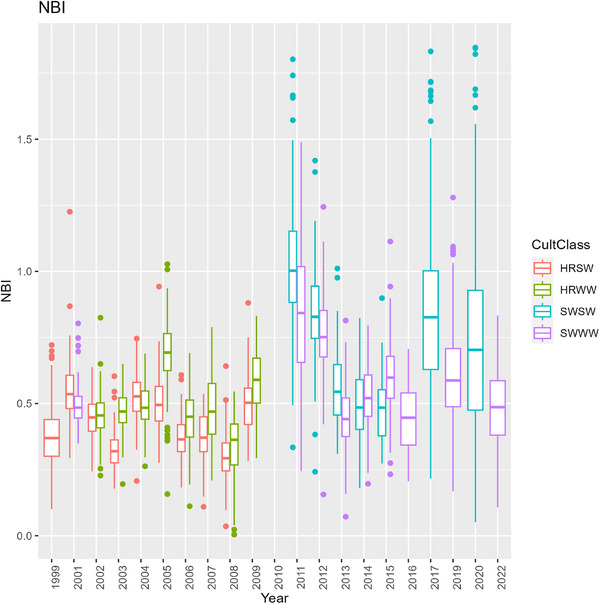
Time series of boxplots of nitrogen balance index (NBI) at Cook Agronomy Farm (CAF), grouped by cultivar class. HRSW, hard red spring wheat; HRWW, hard red winter wheat; SWSW, soft white spring wheat; SWWW, soft white winter wheat.

**FIGURE 8:**
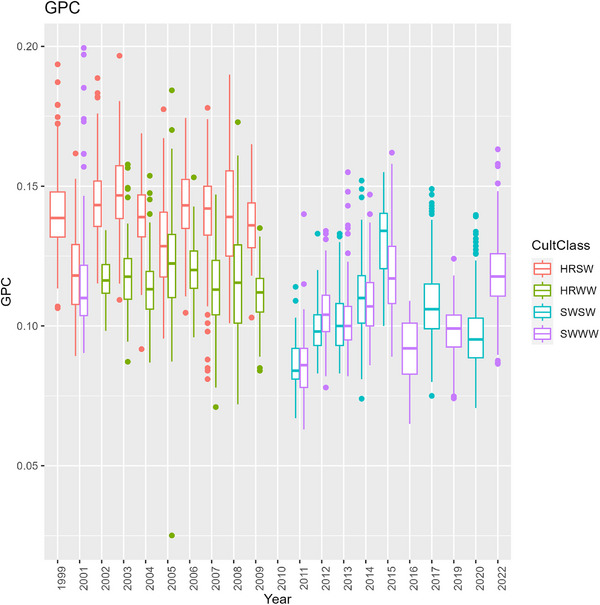
Time series of boxplots of grain protein concentration (GPC) at Cook Agronomy Farm (CAF), grouped by cultivar class. HRSW, hard red spring wheat; HRWW, hard red winter wheat; SWSW, soft white spring wheat; SWWW, soft white winter wheat.

In the first half of CAF's history, wheat was predominantly hard red cultivars (Figure 2), and tended to have a low NBI around 0.5 (Figure 7). There was large variation across the field, nearly 0.1–1.8 at the extreme. Despite inefficient fertilizer use, HRWW mostly had protein levels between 0.10 and 0.12, at or near the target range. HRSW was under target protein levels in many cases (Figure 8). Combined with low NBI values, this could indicate that there are N losses, such that available N is not being used for grain protein accumulation. This is confirmed by examining the ratio of total plant N to N_f_, when there was sufficient data to compute it. It was lower than 1, except in 2017 when following garbanzos, and it was overall higher for soft wheat than hard. A consequence of inefficient N use has been seen in the historic trend at CAF toward declining soil pH (Davis et al., 2023). In the second half of CAF's history, a management decision was made to increase efficiency by switching to soft white cultivars (Figure 2).

Cultivar class has a major impact in managing environmental effects. Brown et al. (2017) found a greater reduction in estimated CO_2_ emission by switching cultivar class than by adopting prescription N_f_. NUpE is lower (around 0.5) in hard red than in soft white (up to 0.78) (Fiez et al., 1995; Huggins et al., 2010; Sowers et al., 1994) but considerable genetic variation exists in both (Latshaw et al., 2016; Van Sanford & MacKown, 1986). This includes genetic variation in the plant ability to remobilize leaf and stem N, take up N after anthesis, but is primarily variation in ability to take up soil N.

NBI improved following the switch to soft white wheat cultivars and reached field means as high as 1.0 in some years, indicating favorable use of applied N. However, NBI for soft white cultivars was more variable, with field means as low as 0.5 in 2013–2016. NBI also exceeded 1 in some instances, indicating crop utilization of both fertilizer and endogenous N sources. While NBI values below 1 are of concern because they indicate environmental loss of N, or cultivars with poor NUpE, NBI values that are above 1 for extended periods could also be of concern if they result from mining of soil organic matter, and utilization of N pools in excess of what are supplied by fertilizer, N‐fixation, and crop residues (McLellan et al., 2018). Coincident with high fertilizer efficiencies, protein concentration for the soft white cultivars also exceeded the narrow, desired range in many cases. In this situation, reducing applied N may have been warranted for reaching production goals and reducing environmental losses.

High NBI in 2011 corresponded to preceding garbanzo bean crops, and in 2017 and 2020 it corresponded to both high Precip_2_ and garbanzos in the preceding 2 years. High precipitation contributed to larger garbanzo yields, greater biomass, and presumably to greater rates of N fixation. This pattern of high NBI following garbanzos demonstrates how strategic rotation of legumes can reduce N losses to the environment and increase yield.

**FIGURE 9:**
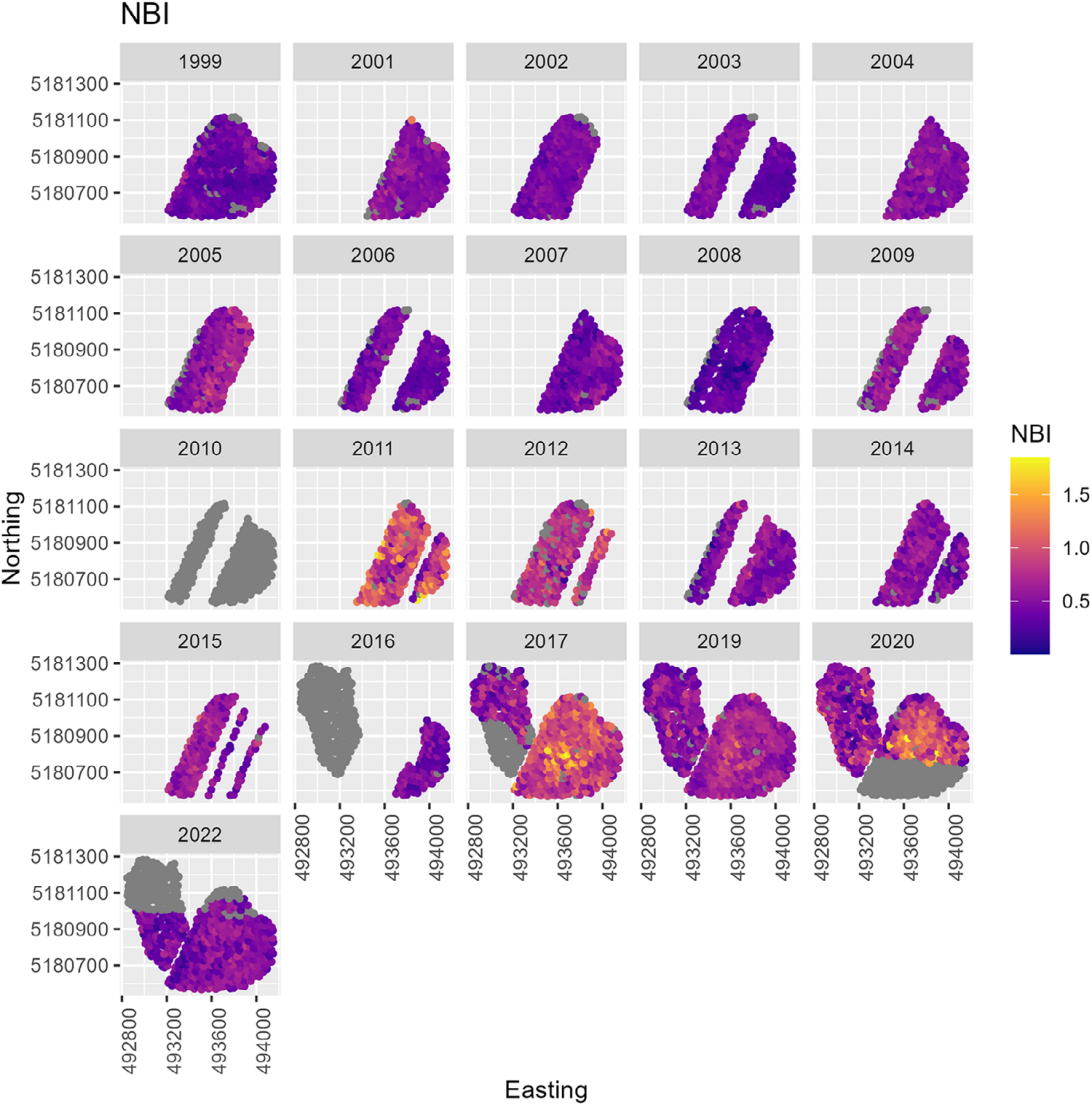
N balance index (NBI) over space and time. Note that hard red wheat (high protein concentration) was grown during 1999–2009, and soft white wheat (low protein concentration) was grown during 2010–2022.

**FIGURE 10:**
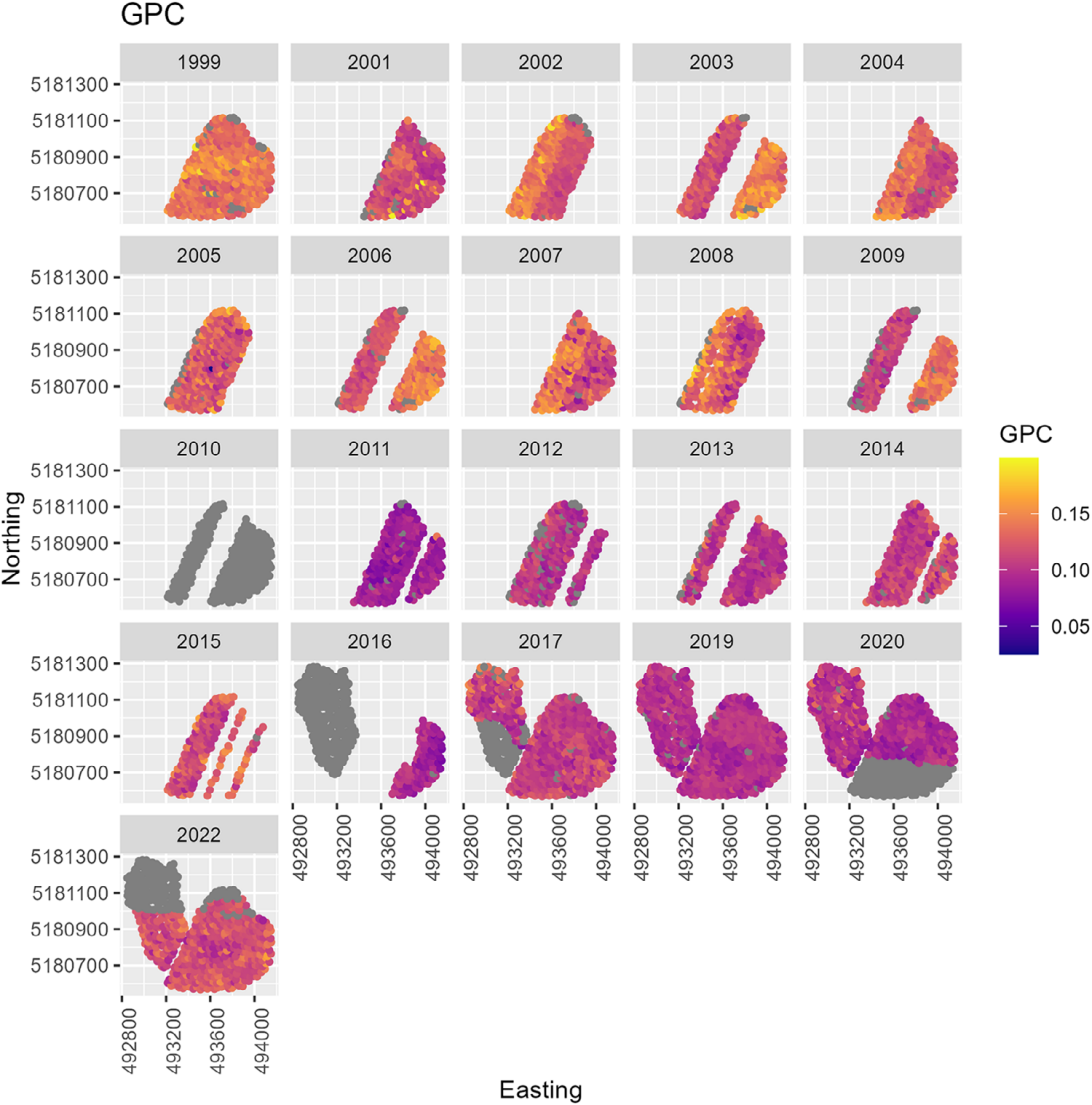
Grain protein concentration (GPC) over space and time.

Across all crop years, determining patterns was challenging due to the changes in cultivar, rotation, and management. Within each crop year, the spatial patterns in NBI and GPC revealed some patterns (Figures 9 and 10). In 1999, for example, high NBI in the Northeast region of Cook East was due to the Palouse type soils in this area, which have a deep rooting depth and no restrictive argillic (Bt) horizon. An Argillic horizon restricts drainage and promotes anaerobic conditions, which favor denitrification, and it can restrict rooting depth (Breslauer et al., 2020; Friedl et al., 2021; Kelley et al., 2017). In 2019, this is also apparent. In 2017—2022, the central region of the East field constituted a lower N rate zone than the surrounding field and consequently shows a higher NBI due to lower N_f_ and crop utilization of accumulated soil N. GPC tended to be higher in this area, except in years with the low‐rate zone. Poorly performing regions of Cook West along the outer edge, and central ridge, corresponded to highly eroded areas. In 2022, high weed pressure and over fertilization resulted in lower NBI.

**FIGURE 11:**
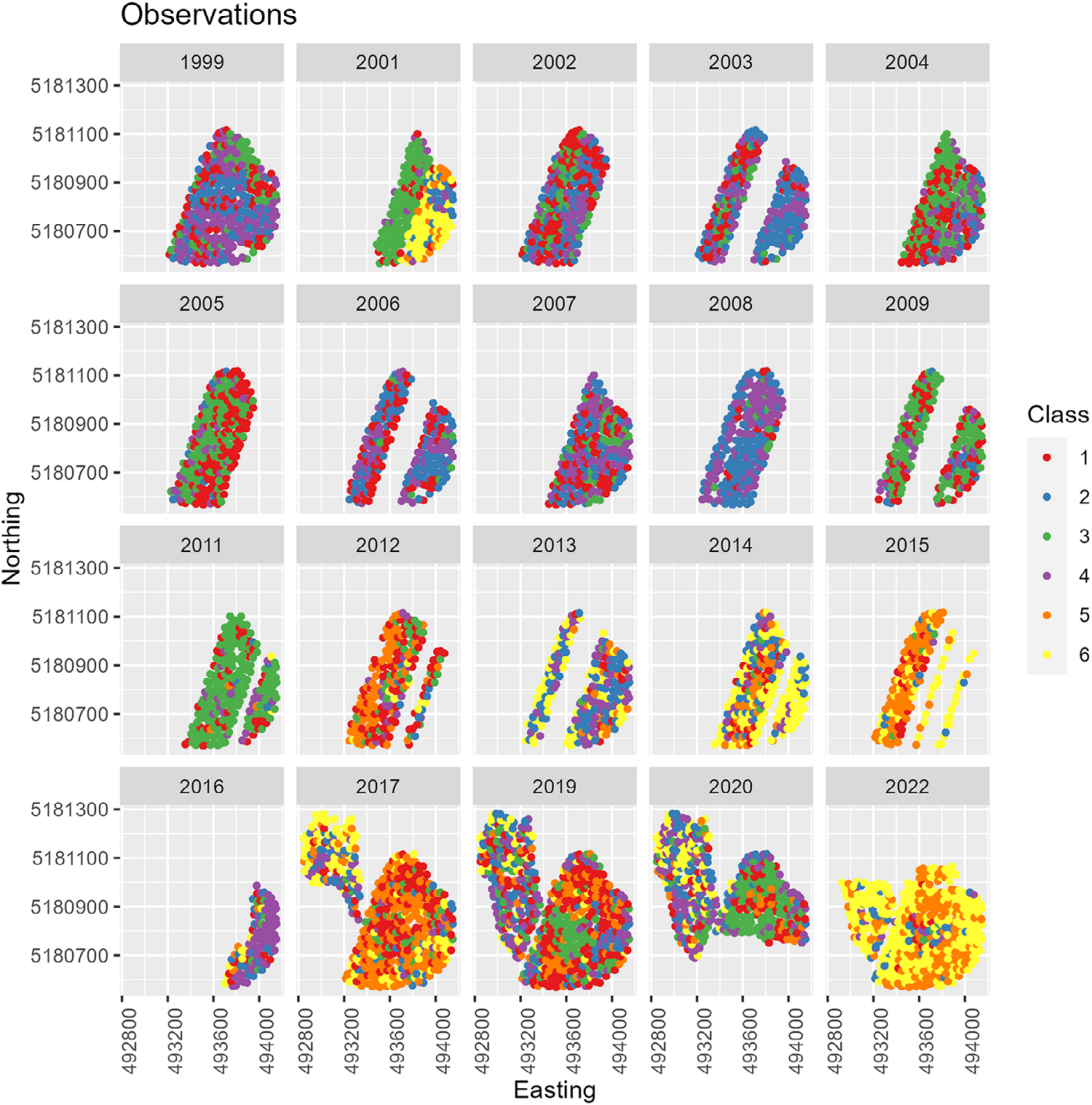
Maps of observed performance class Cook Agronomy Farm (CAF). Only wheat points with sufficient data to calculate the performance class are plotted.

The spatial trends in NBI and GPC were reflected in the classification (Figure 11), although there is a large amount of variability within a harvest year and between years. Hard red cultivars tended to be in Classes 1, 2, or 3 (optimal protein high efficiency, optimal protein/inefficient fertilizer use, or low protein/efficient fertilizer use). Soft white tended to be in Classes 1, 5, or 6 (optimal protein/efficient fertilizer use, high protein/efficient fertilizer use, or high protein/inefficient fertilizer use). In one season (2005), the HRWW managed to achieve the ideal class in most locations, possibly due to lower total N_f_, a result of a recommendation from a lower yield goal. By comparison, in 2008, a similar year with respect to average temperature but higher precipitation and N_f_, was in Classes 2 and 4. NBI could have been lowered by higher N_f_ and uptake being limited by other stressors. Further, 2005 benefitted from high N_f_ in 2004 that left residual N in the soil. Overall, the inter‐season variability, and frequent occurrence of Classes 2, 4, and 6, highlights the influence of other limiting factors that can limit N uptake.

### 3.3 Interpreting N‐performance classes for decision‐making

Table 2 shows each of the classes and corresponding interpretations. Class 2, with adequate protein and low efficiency, is the result of reaching potential yield but using an N rate based on a higher yield goal. This indicates the crop yield is limited by factors besides N, such as soil pH. In this case, the wheat will not make use of all applied N, which is then vulnerable to N losses such as leaching and denitrification. Class 3, with high efficiency and low protein, could improve by applying more N. This is valid to a point, but risks saturating the protein response and reducing efficiency. Such a problem is more likely in soft white wheat, which requires less N per bushel to meet production goals. Class 3 may occur if a producer decides to apply less N to an area, treating it as a lost cause because it is historically low yielding. Then, the area experiences transient conditions favoring growth, but has insufficient N. For example, a waterlogged area in a dry year will have better yield simply due to weather but receives inadequate N application for grain protein accumulation. Class 4, with low efficiency and protein, is the result of N stress limiting growth and protein formation, or anoxic soil conditions due to wet conditions that favor N loss. Increasing applied N might not help, but a producer might try changing the cultivar class, N form, timing of application, fertilizer placement, use of stabilizers, or crop rotations. Multiple factors influence environmental losses of N, notably timing of application relative to precipitation influences denitrification, volatilization, and leaching; form influences acidifying potential; and placement influences availability to the crop (Whetton et al., 2022). An increase in efficiency can be by any of these measures. Class 5 has efficient fertilizer use, which suggests that fertilizer rates could be decreased to reach protein goals and move to Class 1. Higher than necessary fertilizer, though taken up by the plant, may still acidify the soil. Class 6 has inefficient fertilizer use but has high protein, suggesting N more than plant demand was applied. The wheat could have been limited by heat, moisture, or other nutrients, such that high protein occurred because of low yield (T. M. Maaz et al., 2018). Decreasing fertilizer rates may improve efficiency by reducing protein levels, moving the location to Class 1. However, if it was low yielding, this action may neglect the root cause.

**TABLE 2:** Interpretation of performance classes.

Class	Interpretation	Potential environmental effects	Remedies
1	Enough N was applied to meet crop demands. Yield goal probably met.	There is still a possibility of environmental impact through soil mining.	Possibly shifting to soft white cultivar to avoid soil mining.
2	Low NBI is the product of adequate concentration of protein and of low yields. Something else may be limiting besides N. Yield goal probably met.	Unused N could leach or volatilize. Could indicate other environmental limiting factors, such as soil acidity.	Look for other limiting factors, including other nutrients, low organic matter, moisture, or temperature.
3	Efficient N use but low protein indicates N supply was limited during grain accumulation. Low protein indicates yield goal probably not met.	Possible soil mining.	Increased applied N, or making N more available through other nutrients or timing, may raise protein. Consider if efficiency was due to temporary changes in weather or management.
4	Inefficient N use and low protein could mean yield and protein were both low due to N stress.	Could indicate other environmental limiting factors, such as soil acidity.	Look for other limiting factors, including other nutrients, low organic matter, moisture, or temperature. N management needs to change.
5	High protein is a result of excess N. Still a possibility of soil mining. Transient conditions may have favored growth.	Application of excess N, depending on form, could acidify the soil even if it is being used, or result in other environmental losses.	Reduce N application and be mindful of soil N supply.
6	Growth limited by other factors than N. High concentration of protein is a result of low yields.	Unused N could leach or volatilize. Could indicate other environmental limiting factors, such as soil acidity.	Look for other limiting factors, including other nutrients, low organic matter, moisture, or temperature. Consider reducing N rates.

Abbreviation: NBI, nitrogen balance index.

This system is currently undergoing development with producers in the region to develop models using publicly available remotely sensed data to estimate yield, GPC, and N‐performance class. The models capture large‐scale trends over the field, lending the method to generating corrective measures. The goal is to provide a web interface which gives a map of performance class given a few key pieces of management information, including planting and harvest dates, cultivar class, and applied N. The categorical framework has particular benefits for producers in diagnosing particular environmental effects over other systems. The EU Nitrogen Expert Panel (2015) framework of N input versus N output does establish a safe operating zone but makes it harder to gain actionable insights because it is not crop, cultivar class, or site‐specific. The six‐class system allows diagnosing the same environmental issues as looking at GPC, yield, and N_f_, or NUpE and NUtE as in Huggins et al. (2010), but requires less information from producers. It also lends itself well to remote sensing as most producers lack both yield and protein monitors, nor have the time to process and analyze the data.

## 4 CONCLUSION

This paper described and evaluated a novel method for holistic evaluation of wheat performance in the iPNW using data from a long‐term research site. By classifying by protein level and N efficiency, a given point in a wheat crop can be described by its performance class. A multi‐objective metric like this permits a simple evaluation if N application rate, timing, placement, or form needs to be amended to improve returns and reduce N losses through leaching or N_2_O emissions. The multiclass system simplifies the careful balance between market protein levels, reaching potential yield, and efficient N use. This approach differs from past work in its capacity to narrow the range of possible causes of poor N performance on a site‐specific level. Future work will include testing this approach at other sites and implementation in a web app for widespread use among growers and researchers.

## AUTHOR CONTRIBUTIONS

**Joaquin J. Casanova**: Conceptualization; data curation; formal analysis; writing—original draft; writing—review and editing. **David R. Huggins**: Conceptualization; methodology; writing—review and editing. **Claire L. Phillips**: Writing—review and editing.

## CONFLICT OF INTEREST STATEMENT

The authors declare no conflicts of interest.
